# Risk factors for postoperative delirium following total hip or knee arthroplasty: A meta-analysis

**DOI:** 10.3389/fpsyg.2022.993136

**Published:** 2022-09-30

**Authors:** Jinlong Zhao, Guihong Liang, Kunhao Hong, Jianke Pan, Minghui Luo, Jun Liu, Bin Huang

**Affiliations:** ^1^The Second Clinical Medical College of Guangzhou University of Chinese Medicine, Guangzhou, China; ^2^The Second Affiliated Hospital of Guangzhou University of Chinese Medicine, Guangzhou, China; ^3^The Research Team on Bone and Joint Degeneration and Injury of Guangdong Provincial Academy of Chinese Medical Sciences, Guangzhou, China; ^4^Guangdong Second Traditional Chinese Medicine Hospital (Guangdong Province Engineering Technology Research Institute of Traditional Chinese Medicine), Guangzhou, China; ^5^The Fifth Clinical Medical College of Guangzhou University of Chinese Medicine, Guangzhou, China

**Keywords:** post-operative delirium, risk factor, systematic review, total joint arthroplasty, meta-analysis

## Abstract

**Objectives:**

The purpose of this study was to identify risk factors for delirium after total joint arthroplasty (TJA) and provide theoretical guidance for reducing the incidence of delirium after TJA.

**Methods:**

The protocol for this meta-analysis is registered with PROSPERO (CRD42020170031). We searched PubMed, the Cochrane Library and Embase for observational studies on risk factors for delirium after TJA. Review Manager 5.3 was used to calculate the relative risk (RR) or standard mean difference (SMD) of potential risk factors related to TJA. STATA 14.0 was used for quantitative publication bias evaluation.

**Results:**

In total, 25 studies including 3,767,761 patients from 9 countries were included. Old age has been widely recognized as a risk factor for delirium. Our results showed that the main risk factors for delirium after TJA were patient factors (alcohol abuse: RR = 1.63; length of education: SMD = −0.93; and MMSE score: SMD = −0.39), comorbidities (hypertension: RR = 1.26; diabetes mellitus: RR = 1.67; myocardial infarction: RR = 17.75; congestive heart failure: RR = 2.54; dementia: RR = 17.75; renal disease: RR = 2.98; history of stroke: RR = 4.83; and history of mental illness: RR = 2.36), surgical factors (transfusion: RR = 1.53; general anesthesia: RR = 1.10; pre-operative albumin: SMD = −0.38; pre-operative hemoglobin: SMD = −0.29; post-operative hemoglobin: SMD = −0.24; total blood loss: SMD = 0.15; duration of surgery: SMD = 0.29; and duration of hospitalization: SMD = 2.00) and drug factors (benzodiazepine use: RR = 2.14; ACEI use: RR = 1.52; and beta-blocker use: RR = 1.62).

**Conclusions:**

Multiple risk factors were associated with delirium after TJA. These results may help doctors predict the occurrence of delirium after surgery and determine the correct treatment.

**Systematic review registration:**

https://www.crd.york.ac.uk/prospero/, identifier: CRD42020170031.

## Introduction

Total hip arthroplasty (THA) and total knee arthroplasty (TKA) have been shown by a large number of clinical studies to be very successful surgical methods for treating serious hip and knee joint diseases. Total joint arthroplasty (TJA) can significantly improve a patient's quality of life and reduce hip and knee pain (Schiffern et al., 2012; Kong et al., 2017; Petersen et al., 2018). Although TJA has many therapeutic advantages, many clinical complications may occur after surgery. Post-operative delirium (POD) is a clinical complication that seriously affects the quality of life of patients. POD is a post-operative acute mental disorder that is often accompanied by transient disturbances in attention, feelings, thinking, memory, and sleep cycles; attention disorder is the core symptom (Allen and Frankel, 2012). POD is a common central nervous system complication in elderly patients. It can significantly prolong the length of hospital stay, increase the incidence and mortality of dementia, and cause high burdens on patients and the medical system (Allen and Frankel, 2012; Oh and Park, 2019). Studies have shown that the incidence of POD among hospitalized patients is 20–31% (Siddiqi et al., 2006), and the incidence of delirium can exceed 50% in elderly patients undergoing THA (Rizk et al., 2016). The incidence of delirium after TKA in elderly patients can reach as high as 48% (Kinjo et al., 2012). POD increases the risk of death in patients. For every 48 h of delirium symptoms, the risk of death increases by 10–20%, but 30–40% of POD can be prevented (Schenning and Deiner, 2015; Hamilton et al., 2017). Therefore, identifying factors associated with a high risk of POD and establishing targeted treatment measures are important in reducing the incidence of delirium and complications caused by POD.

The pathogenesis of POD is still unclear. There are currently many hypotheses, including neurotransmitter disorders, systemic inflammation, and sleep cycle disorders (Maldonado, 2013). Due to the unknown pathogenesis of POD, symptomatic treatment with targeted drugs is lacking in the clinic. In addition, the occurrence and development of POD is not conducive to lower limb function training and rehabilitation processes in patients with TJA, potentially leading to post-operative cognitive impairment.

Therefore, surgeons should be aware of the risk of POD and manage patients with associated risk factors appropriately. The purpose of this meta-analysis and systematic literature review is to quantitatively analyze risk factors for POD and provide an effective theoretical basis for the prevention of POD.

## Methods

This meta-analysis was performed in strict accordance with the relevant requirements of the Meta-analysis of Observational Studies in Epidemiology (MOOSE) statement (Stroup et al., 2000). This study was registered at the International Prospective Register of Systematic Reviews (Registration Number CRD42020170031).

### Inclusion and exclusion criteria

The inclusion criteria were as follows: (1) published observational studies, including case–control studies, retrospective cohort studies, and prospective cohort studies; (2) studies involving patients who underwent TKA or THA, regardless of age, sex or nationality; (3) studies in which the intervention was TKA or THA, not limited to initial or revision surgery; (4) studies in which the results involved POD; (5) studies that analyzed data on risk factors for delirium after TJA; and (6) studies that were published in only the English language.

The exclusion criteria were as follows: (1) reviews, meeting abstracts, and case reports; (2) duplicate publications or studies with identical data; and (3) studies that did not have sufficient data to calculate means and SDs and for which the data were not available to the authors.

### Literature retrieval strategy

The PubMed, Cochrane Library and Embase databases were searched, and observational studies meeting the inclusion criteria were included. The retrieval time was from the establishment of each database to June 2022. See Supplementary material 1 for the retrieval strategy for each database. We also manually searched all the references of the included studies to identify other studies that might be eligible for inclusion.

### Literature screening and data extraction

Two orthopedic surgeons retrieved the literature, and preliminary and secondary screening of the literature was conducted in strict accordance with the pre-established inclusion and exclusion criteria. Two researchers independently extracted the data, and a third researcher carried out the comparisons. In cases of errors or differences, the third researcher and corresponding author assisted in the judgment.

The data extracted in this study included the title, first author, publication year, country, sample size, surgery location (knee, hip), mean age, identified significant risk factors, relevant items for literature quality evaluation and all possible associated risk factors.

### Quality assessment of the included studies

The Newcastle–Ottawa Scale (NOS) was used to evaluate the quality of the included observational literature. The NOS evaluation includes 4 items (4 points) to evaluate selection, 1 item (2 points) to evaluate the comparability of groups, and 3 items (3 points) to evaluate the outcome of interest. The highest possible score is 9 points. Studies with a score ≥ 6 are considered high quality, and those with a score <6 are considered low quality. The NOS uses a semiquantitative star system to evaluate the quality of the literature. If a study meets the standard, it receives 1 star per item, with a total score of 9 points. The higher the score is, the higher the quality of the literature (Lo et al., 2014). Two researchers independently completed the quality evaluations of the included studies. If there were inconsistencies, they were resolved by consultation with a corresponding author.

### Statistical analysis

Relative risks (RRs) were used to evaluate the effects of binary variables, and standard mean differences (SMDs) were used to evaluate the effects of continuous variables; 95% confidence intervals (CIs) of the RRs and SMDs were calculated. Review Manager 5.3.5 software (Cochrane Collaboration, Oxford, UK) was used to calculate the efficacy and safety indicators and their 95% CIs. In addition, for homogeneous datasets, *P* > 0.1 and *I*^2^ < 50% were considered the test standards. When the above two statistical conditions were met, a fixed-effect model was used for the meta-analysis because the pooled effect sizes were relatively homogenous. If one of the above standards did not conform, the homogeneity of the pooled effect size was not ideal, and the random effects model was applied. For factors with a significant difference, an RR ≥ 2 was considered high risk, 1 < RR < 2 was considered medium risk, and an RR < 1 was considered a protective factor.

When both THA and TKA were investigated, a subgroup analysis was performed according to surgical site, but the condition was that the same subgroup must include 2 or more studies. To quantitatively assess whether there was publication bias in the different risk factor indicators, Stata 14.0 (STATA Corporation, Lakeway, Texas, USA) software was used to perform Egger's and Begg's linear regression tests on the outcome indicators that were included in the combined analysis of 10 or more studies.

## Results

### Literature screening process and results

**Figure 1:**
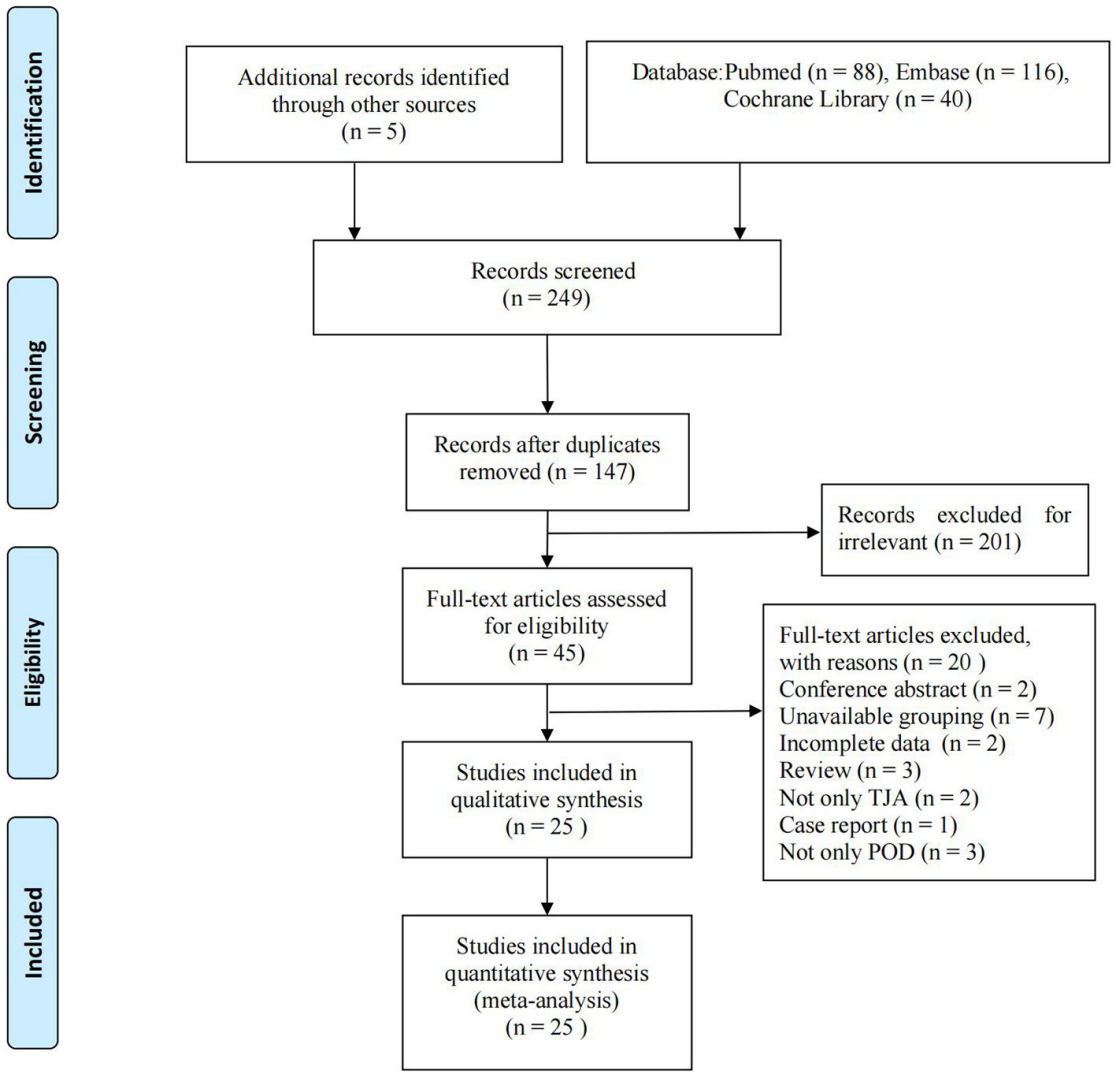
Flow diagram details the process of relevant clinical study selection.

A total of 249 relevant documents were obtained during the preliminary searches of the PubMed (*n* = 88), Embase (*n* = 116), and Cochrane Library (*n* = 40) databases and other manual searches (*n* = 5). After reading the titles and abstracts and excluding irrelevant literature, 45 articles were retained. After excluding duplicate studies and applying the inclusion criteria and exclusion criteria, 25 studies (Priner et al., 2008; Jankowski et al., 2011; Flink et al., 2012; Nandi et al., 2014; Chung et al., 2015; Wang et al., 2016, 2022; Yen et al., 2016; Bosmak et al., 2017; Chen et al., 2017, 2021a,b; Cunningham et al., 2017, 2019; Petersen et al., 2017; Aziz et al., 2018; Weinstein et al., 2018; Huang et al., 2019; Memtsoudis et al., 2019; Peng et al., 2019; He et al., 2020; Kijima et al., 2020; Qi et al., 2020; Jiang and Lei, 2022; Lin et al., 2022) including 3,767,761 patients from 9 countries were finally included. One study (Memtsoudis et al., 2019) separately reported the risk factors for delirium after TKA and THA. The literature screening process and results are shown in Figure 1. The basic information of the included literature is shown in Table 1.

**Table 1:** Characteristics of the included studies.

**References**	**Country**	**Study design**	**Period**	**Sample size**		**Gender (M/F)**				**Age, years**		**Position**	**NOS**
				**POD**	**No-POD**	**POD**		**No-POD**		**POD**	**No-POD**		
Memtsoudis et al., 2019	USA	CO, R	2006–2016	14,785	549,441	5,964	8,821	244,936	304,505	69	65	Hip	9
Memtsoudis et al., 2019	USA	CO, R	2006–2016	32,384	1,098,185	11,289	21,095	410,984	687,201	69	66	Knee	9
Bosmak et al., 2017	Brazil	CO, R	2015.4–2015.12	5	52	1	4	23	29	73.4	62.67	Knee, hip	8
Wang et al., 2016	China	CO, R	2011.1–2014.12	75	507	–	–	–	–	–	–	Knee, hip	8
Huang et al., 2019	USA	CO, R	2008.1–2012.12	181	11,789	87	94	5,299	6,490	75.4 ± 11.7	65.9 ± 12.2	Knee, hip	8
Chen et al., 2017	China	CO, P	2014.8–2015.5	35	177	10	25	45	132	81.8 ± 4.9	72.2 ± 5.1	Knee, hip	7
Chung et al., 2015	Korea	CO, R	2009.4–2013.5	11	354	1	10	32	322	75	71	Knee	8
Aziz et al., 2018	USA	CO, R	2000–2009	13,551	1,992,971	7,589	5,962	1,036,345	956,626	75 ± 0.2	65 ± 0.1	Hip	9
Cunningham et al., 2019	UK	CO, P	–	40	242	19	21	104	138	76.6 ± 6.0	73.8 ± 5.6	Knee, hip	9
Jankowski et al., 2011	USA	CO, P	–	42	376	23	19	183	193	74.76 ± 6.07	72.74 ± 5.31	Knee, hip	8
Nandi et al., 2014	USA	C-C	2006–2010	98	365	44	54	165	200	–	–	Knee, hip	8
Flink et al., 2012	USA	CO, P	–	27	79	9	18	38	41	72.9 ± 4.9	73.7 ± 5.1	Knee	8
Cunningham et al., 2017	UK	CO, P	–	40	275	18	22	118	157	76.9 ± 6	74.0 ± 5.7	Knee, hip	8
Peng et al., 2019	China	CO, P	2015.3–2018.3	55	217	22	33	93	124	74.5 ± 5.6	72.1 ± 6.1	Knee, hip	8
Petersen et al., 2017	Denmark	CO, P	2010.2–2013.11	43	6,288	21	22	2,371	3,917	80.7	76.68	Knee, hip	7
Weinstein et al., 2018	USA	CO, R	2005–2014	922	40,844	341	581	16,816	24,028	78	66	Knee, hip	9
Qi et al., 2020	China	CO, R	2016.10–2019.1	68	260	27	41	105	155	72.4 ± 4.1	72.1 ± 3.7	Knee, hip	8
Priner et al., 2008	France	CO, P	–	15	86	–	–	–	–	78.2 ± 4.4	72.8 ± 6.6	Knee, hip	7
Yen et al., 2016	Singapore	CO, P	–	22	76	9	13	38	38	72.5 ± 4.4	73.7 ± 5.2	Knee	7
He et al., 2020	China	CO, P	2014.3–2019.12	182	598	83	99	296	302	75.77 ± 8.57	73.28 ± 7.44	Hip	8
Chen et al., 2021b	China	CO, R	2013.1–2019.10	67	927	18	49	268	659	71.1 ± 9.6	66.4 ± 9.7	Knee, hip	9
Jiang and Lei, 2022	China	CO, R	2018–2021	43	293	18	25	122	171	74.3 ± 3.1	72.1 ± 2.9	Knee, hip	8
Chen et al., 2021a	China	CO, R	2017.1–2020.5	65	195	16	49	51	144	85.06 ± 7.04	82.77 ± 8.29	Knee, hip	8
Wang et al., 2022	China	CO, P	2020.6–2020.11	53	53	31	22	33	20	72.85 ± 6.29	71 ± 5.73	Knee	7
Lin et al., 2022	China	CO, P	–	66	66	33	33	46	20	61	68	Knee, hip	7
Kijima et al., 2020	Japan	CO, R	2013.6–2015.4	11	159	2	9	30	129	79.5 ± 6.9	73 ± 9	Knee	8

Co, Cohort study; P, prospective study; R, retrospective study; C-C, Case-control; M, Male; F, Female; POD, post-operative delirium; NOS, Newcastle-Ottawa Scale.

### Quality evaluation of the included literature

The 25 observational studies included in this meta-analysis included 12 retrospective cohort studies, 12 prospective cohort studies, and 1 case–control study, with literature quality evaluation scores of 7–9. According to the NOS criteria, 5 studies received 9 points, 14 studies received 8 points, and 6 studies received 7 points. Each article included in this study had an NOS score > 6 points, indicating that the quality of the literature was very high (Tables 1, 2).

**Table 2:** Newcastle-Ottawa scale for risk of bias assessment of cohort studies included in the meta-analysis.

**References**	**Selection**				**Comparability**	**Outcome**			**Overall**
	**Representativeness of** **exposed cohort**	**Selection of** **non-exposed**	**Ascertainment of** **exposure**	**Outcome not present** **at start**		**Assessment of** **outcome**	**Adequate follow-up** **length**	**Adequacy of** **follow-up**	
Memtsoudis et al., 2019	⋆	⋆	⋆	⋆	⋆⋆	⋆	⋆	⋆	9
Bosmak et al., 2017	⋆	⋆	⋆	⋆	⋆	⋆	⋆	⋆	8
Wang et al., 2016	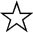	⋆	⋆	⋆	⋆⋆	⋆	⋆	⋆	8
Huang et al., 2019	⋆	⋆	⋆	⋆	⋆	⋆	⋆	⋆	8
Chen et al., 2017	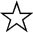	⋆	⋆	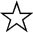	⋆⋆	⋆	⋆	⋆	7
Chung et al., 2015	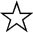	⋆	⋆	⋆	⋆⋆	⋆	⋆	⋆	8
Aziz et al., 2018	⋆	⋆	⋆	⋆	⋆⋆	⋆	⋆	⋆	9
Cunningham et al., 2019	⋆	⋆	⋆	⋆	⋆⋆	⋆	⋆	⋆	9
Jankowski et al., 2011	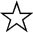	⋆	⋆	⋆	⋆⋆	⋆	⋆	⋆	8
Nandi et al., 2014	⋆	⋆	⋆	⋆	⋆⋆	⋆	⋆	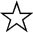	8
Flink et al., 2012	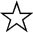	⋆	⋆	⋆	⋆⋆	⋆	⋆	⋆	8
Cunningham et al., 2017	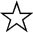	⋆	⋆	⋆	⋆⋆	⋆	⋆	⋆	8
Peng et al., 2019	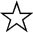	⋆	⋆	⋆	⋆⋆	⋆	⋆	⋆	8
Petersen et al., 2017	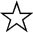	⋆	⋆	⋆	⋆	⋆	⋆	⋆	7
Weinstein et al., 2018	⋆	⋆	⋆	⋆	⋆⋆	⋆	⋆	⋆	9
Qi et al., 2020	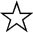	⋆	⋆	⋆	⋆⋆	⋆	⋆	⋆	8
Priner et al., 2008	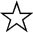	⋆	⋆	⋆	⋆	⋆	⋆	⋆	7
Yen et al., 2016	⋆	⋆	⋆	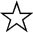	⋆	⋆	⋆	⋆	7
He et al., 2020	⋆	⋆	⋆	⋆	⋆⋆	⋆	⋆	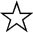	8
Chen et al., 2021b	⋆	⋆	⋆	⋆	⋆⋆	⋆	⋆	⋆	9
Jiang and Lei, 2022	⋆	⋆	⋆	⋆	⋆⋆	⋆	⋆	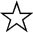	8
Chen et al., 2021a	⋆	⋆	⋆	⋆	⋆	⋆	⋆	⋆	8
Wang et al., 2022	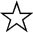	⋆	⋆	⋆	⋆	⋆	⋆	⋆	7
Lin et al., 2022	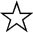	⋆	⋆	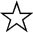	⋆⋆	⋆	⋆	⋆	7
Kijima et al., 2020	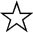	⋆	⋆	⋆	⋆⋆	⋆	⋆	⋆	8

⋆, score of 1; ⋆⋆, score of 2; 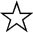, score of 0.

### Findings from the meta-analysis

#### Patient factors

We performed a meta-data analysis of seven influencing patient factors, including gender, smoking, alcohol abuse, body mass index (BMI), race, education level, and mini-mental state examination (MMSE) score (Table 3). Based on the statistical results of the meta-analysis, we identified the following as influencing factors for delirium after TJA: male sex (RR: 0.92, 95% CI: 0.91–0.93), alcohol abuse (RR: 1.63, 95% CI: 1.13–2.36), black race (RR: 0.88, 95% CI: 0.85–0.92), length of education (SMD: −0.93, 95% CI: −1.09 to −0.77) and MMSE (SMD: −0.39, 95% CI: −0.55 to −0.24). Among the significant risk factors, alcohol abuse was the most serious risk factor for delirium after TJA and was associated with high risk. Male sex and black race were protective factors. A low education level and low MMSE score were risk factors for POD. Other factors (BMI and smoking) were not considered to be significant risk factors for delirium after TJA (*P* > 0.05). Since the heterogeneity in age in the meta-analysis was 100% and the source of the heterogeneity could not be identified by sensitivity and meta-regression analyses, we performed a descriptive analysis.

**Table 3:** The main outcomes of meta-analysis and subgroup analysis (patients factors).

**Risk factors**	**No. of studies**	**RR or SMD**	**LL 95%CI**	**UL 95%CI**	***P-*value**	***I^2^* (%)**	**Analysis model**
**Male gender**	23	0.92^*^	0.91	0.93	<0.001	4	M-H, fixed
THA subgroup	3	0.97^*^	0.84	1.13	0.70	99	M-H, random
TKA subgroup	6	0.93^*^	0.92	0.95	<0.001	0	M-H, fixed
**Female gender**	24	1.02^*^	0.98	1.07	0.36	89	M-H, random
THA subgroup	3	1.01^*^	0.88	1.16	0.84	99	M-H, random
TKA subgroup	6	1.04^*^	1.03	1.05	<0.001	0	M-H, fixed
**Smoking**	10	1.02^*^	0.93	1.13	0.65	0	M-H, fixed
**Alcohol abuse**	7	1.63^*^	1.13	2.36	0.009	50	M-H, random
**BMI**	9	−0.09^†^	−0.18	0.01	0.07	33	IV, fixed
TKA subgroup	4	−0.06^†^	−0.29	0.16	0.58	0	IV, fixed
**White race**	5	1.00^*^	0.99	1.02	0.66	82	M-H, random
TKA subgroup	3	0.84^*^	0.77	0.92	0.0001	83	M-H, random
**Black race**	2	0.88^*^	0.85	0.92	<0.001	0	M-H, fixed
**Length of education**	5	−0.93^†^	−1.09	−0.77	<0.001	40	IV, random
TKA subgroup	2	−0.11^†^	−0.43	0.21	0.50	0	IV, fixed
**MMSE**	6	−0.39^†^	−0.55	−0.24	<0.001	46	IV, random
TKA subgroup	2	−0.49^†^	−1.32	0.34	0.25	84	IV, random

THA, total hip arthroplasty; TKA, total knee arthroplasty; BMI, body mass index; MMSE, Mini-mental State Examination; RR, Relative Risk; SMD, standardized mean differences; LL, lower limit; UL, upper limit; M-H, Mantel Haenszel test; IV, inverse variance.

* RR.

† SMD.

The forest map of all risk factors is shown in Supplementary material 2.

We performed an additional subgroup analysis based on the location of surgery (hip or knee). In the TKA subgroup, white race (RR: 0.84, 95% CI: 0.77–0.92) and male sex (RR: 0.93, 95% CI: 0.92–0.95) were protective factors, but female sex was a risk factor (RR: 1.04, 95% CI: 1.03–1.05). In the THA subgroup, the incidence of POD was not significantly different between males and females.

#### Comorbidities

We analyzed the effects of 10 coexisting diseases and past medical history characteristics, including hypertension, diabetes mellitus (DM), obstructive sleep apnea (OSA), myocardial infarction, congestive heart failure (CHF), pulmonary disease, dementia, renal disease, stroke, and history of mental illness, on delirium after TJA (Table 4). Based on the combined RRs, we identified the following risk factors: hypertension (RR: 1.26, 95% CI: 1.12–1.43), DM (RR: 1.67, 95% CI: 1.52–1.83), myocardial infarction (RR: 3.21, 95% CI: 2.74–3.76), CHF (RR: 2.54, 95% CI: 1.77–3.64), dementia (RR: 17.75, 95% CI: 9.84–32.01), renal disease (RR: 2.98, 95% CI: 1.93–4.62), history of stroke (RR: 4.83, 95% CI: 2.33–10.01), and history of mental illness (RR: 2.36, 95% CI: 2.32–2.41). Among all the significant risk factors, myocardial infarction, CHF, dementia, renal disease, stroke and history of mental illness were associated with high risks, and dementia was associated with the highest risk of delirium after TJA. OSA and pulmonary disease were not significant risk factors for delirium after TJA (*P* > 0.05).

**Table 4:** The main outcomes of meta-analysis and subgroup analysis (comorbidities).

**Risk factors**	**No. of studies**	**RR**	**LL 95%CI**	**UL 95%CI**	***P-*value**	***I^2^* (%)**	**Analysis model**
**Hypertension**	12	1.26	1.12	1.43	0.0002	66	M-H, random
TKA subgroup	3	1.41	1.21	1.65	<0.001	68	M-H, random
**Diabetes mellitus**	13	1.67	1.52	1.83	<0.001	45	M-H, fixed
TKA subgroup	3	1.66	0.89	3.09	0.11	0	M-H, fixed
**Obstructive sleep apnea**	3	0.97	0.83	1.12	0.64	0	M-H, fixed
TKA subgroup	2	3.40	1.77	6.53	0.0002	0	M-H, fixed
**Myocardial infarction**	4	3.21	2.74	3.76	<0.001	81	M-H, random
**Congestive heart failure**	2	2.54	1.77	3.64	<0.001	0	M-H, fixed
**Pulmonary disease**	2	1.20	0.89	1.62	0.22	64	M-H, random
**Dementia**	3	17.75	9.84	32.01	<0.001	0	M-H, fixed
**Renal disease**	4	2.98	1.93	4.62	<0.001	75	M-H, random
**History of stroke**	3	4.83	2.33	10.01	<0.001	65	M-H, random
**History of mental illness**	6	2.36	2.32	2.41	<0.001	0	M-H, fixed

RR, Relative Risk; SMD, standardized mean differences; THA, total hip arthroplasty; TKA, total knee arthroplasty; LL, lower limit; UL, upper limit; M-H, Mantel Haenszel test; IV, inverse variance.

The forest map of all risk factors is shown in Supplementary material 2.

In the TKA subgroup, hypertension (RR: 1.41, 95% CI: 1.21–1.65) and OSA (RR: 3.4, 95% CI: 1.77–6.53) were factors associated with high risk. DM was not a risk factor for delirium after TKA (*P* > 0.05). Due to the lack of data on hip surgeries, we did not conduct a subgroup analysis according to the location of surgery.

#### Surgical factors

This meta-analysis analyzed the effects of 8 surgical factors, including transfusion, type of anesthesia (general or spinal), duration of hospitalization, pre-operative albumin and hemoglobin, post-operative hemoglobin, duration of surgery, and total blood loss, on delirium after TJA (Table 5). The following were found to be influencing factors: transfusion (RR: 1.53, 95% CI: 1.16–2.00), general anesthesia (RR: 1.10, 95% CI: 1.06–1.14), spinal anesthesia (RR: 0.85, 95% CI: 0.83–0.88), duration of hospitalization (SMD: 2.00, 95% CI: 1.97–2.03), pre-operative albumin (SMD: −0.38, 95% CI: −0.50 to −0.26), pre-operative hemoglobin (SMD: −0.29, 95% CI: −0.54 to −0.04), post-operative hemoglobin (SMD: −0.24, 95% CI: −0.44 to −0.04), duration of surgery (SMD: 0.29, 95% CI: 0.18–0.40) and total blood loss (SMD: 0.15, 95% CI: 0.02–0.29). In TJA patients, transfusion and spinal anesthesia were protective factors, and general anesthesia, a long operation time, a long hospital stay, low albumin and hemoglobin before surgery, blood loss and low hemoglobin after surgery were all risk factors for delirium.

**Table 5:** The main outcomes of meta-analysis and subgroup analysis (surgical factors).

**Risk factors**	**No. of studies**	**RR or SMD**	**LL 95%CI**	**UL 95%CI**	***P-*value**	***I^2^* (%)**	**Analysis model**
**Transfusion**	5	1.53^*^	1.16	2.00	0.002	0	M-H, fixed
**Type of anesthesia (General)**	6	1.10^*^	1.06	1.14	<0.001	84	M-H, random
TKA subgroup	2	0.93^*^	0.60	1.46	0.76	53	M-H, random
**Type of anesthesia (Spinal)**	5	0.85^*^	0.83	0.88	<0.001	35	M-H, fixed
TKA subgroup	2	1.01^*^	0.71	1.43	0.96	84	M-H, random
**Duration of hospitalization**	3	2.00^†^	1.97	2.03	<0.001	15	I-V, fixed
**Pre-operative Albumin (g/L)**	5	−0.38^†^	−0.50	−0.26	<0.001	49	I-V, fixed
**Pre-operative Hemoglobin (g/L)**	4	−0.29^†^	−0.54	−0.04	0.02	69	I-V, random
**Post-operative Hemoglobin (g/L)**	2	−0.24^†^	−0.44	−0.04	0.02	0	I-V, fixed
**Duration of surgery (minutes)**	8	0.25^†^	0.12	0.38	0.0002	52	I-V, random
**Total blood loss (ml)**	6	0.15^†^	0.02	0.29	0.03	20	I-V, fixed

RR, Relative Risk; SMD, standardized mean differences; THA, total hip arthroplasty; TKA, total knee arthroplasty; LL, lower limit; UL, upper limit; M-H, Mantel Haenszel test; I-V, inverse variance.

* RR.

† SMD.

The forest map of all risk factors is shown in Supplementary material 2.

In the TKA subgroup, neither general anesthesia (RR: 0.93, 95% CI: 0.60–1.46) nor spinal anesthesia (RR: 1.01, 95% CI: 0.71–1.43) were risk factors for POD.

#### Drug factors

We evaluated the risk for delirium after TJA considering the use of 6 drugs, including sustained-release oxycodone, benzodiazepines, ketamine, angiotensin-converting enzyme inhibitors (ACEIs), beta-blockers, and statins (Table 6). Based on the combined RRs, benzodiazepine (RR: 2.14, 95% CI: 1.87–2.45), ACEIs (RR: 1.52, 95% CI: 1.02–2.27) and beta-blocker use (RR: 1.62, 95% CI: 1.19–2.21) were high risk factors for delirium after TJA. Sustained-release oxycodone, ketamine and statin use were not risk factors (*P* > 0.05).

**Table 6:** The main outcomes of meta-analysis and subgroup analysis (drug factors).

**Risk factors**	**No. of studies**	**RR**	**LL 95%CI**	**UL 95%CI**	***P-*value**	***I^2^* (%)**	**Analysis model**
Sustained-release oxycodone	2	0.77	0.59	1.01	0.06	0	M-H, fixed
Benzodiazepines	2	2.14	1.87	2.45	<0.001	0	M-H, fixed
Ketamine	3	1.01	0.97	1.06	0.45	51	M-H, random
ACEIs	3	1.52	1.02	2.27	0.04	0	M-H, fixed
β-blockers	3	1.62	1.19	2.21	0.002	0	M-H, fixed
Statins	2	1.01	0.57	1.80	0.97	0	M-H, fixed

RR, Relative Risk; THA, total hip arthroplasty; TKA, total knee arthroplasty; LL, lower limit; UL, upper limit; M-H, Mantel Haenszel test; I-V, inverse variance.

The forest map of all risk factors is shown in Supplementary material 2.

#### Evaluation of publication bias

To quantitatively analyze whether there was publication bias in the relevant outcome indicators of this study, we conducted Egger's and Begg's tests on the outcome indicators included in 10 or more studies. Begg's tests suggested no significant publication bias in the following influencing factors: male sex (*P* = 0.316), female sex (*P* = 0.291), smoking (*P* = 0.474), DM (*P* = 0.951) and hypertension (*P* = 0.5372). The data analysis process and statistical results of the publication bias analysis are shown in Supplementary material 3.

## Discussion

Identifying risk factors and taking appropriate preventive measures can prevent and reduce POD (Schenning and Deiner, 2015; Hamilton et al., 2017), and a comprehensive and systematic understanding of risk factors for POD after TJA is necessary. This meta-analysis analyzed 31 related factors in four categories (patient factors, comorbidities, surgical factors, and drug factors) and evaluated the impact of these factors on delirium after TJA.

### Patient factors

Alcohol abuse was associated with the highest risk for delirium after TJA. Our meta-analysis results indicated that among the patient-related factors, alcohol abuse was a direct cause of delirium in patients after TJA. The results of a large cohort study suggest that alcohol plays a key role in the development of delirium in patients (Van Rompaey et al., 2009). Alcohol abuse was considered an independent risk factor for POD in elderly patients in previous studies (Sousa et al., 2017; Stewart et al., 2019), consistent with our findings. Mukherjee S believes that long-term alcohol abuse slows metabolism in the body, causing the content of ethanol and its metabolite acetaldehyde in the blood to increase sharply; because of the lipophilicity of the blood–brain barrier, the two compounds easily pass through the barrier and cause direct damage to nerve tissue (Mukherjee, 2013). Therefore, long-term alcohol abuse can cause changes in the structure of brain cells, causing the hippocampus, which has important cognitive functions such as learning and memory, to lose a large number of glial cells, resulting in brain atrophy and cognitive dysfunction manifesting as gradual intellectual decline and memory loss and even personality changes (Mukherjee, 2013). Controlling or eliminating alcohol intake in patients who undergo TJA plays a key role in preventing POD. A low education level and low MMSE score were associated with a high risk of POD; these results may be related to the poor attention given by patients to their own health status and an insufficient understanding of surgery and anesthesia (Lee et al., 2011; Grover et al., 2016). Male sex and black race were associated with relatively low risks of POD after TJA. In addition, our study found that BMI and smoking were not risk factors for delirium after TJA. In the TKA subgroup, white race and male sex were associated with low risks of POD. In the THA subgroup, sex was not a risk factor for POD. After TKA, there is still a lack of research on the causes and mechanisms of female sex (male sex is a protective factor) pre-disposing to POD, which requires further exploration and proper explanation in future animal experiments and clinical studies.

The heterogeneity in age in the meta-analysis was 100%, and the sensitivity and meta-regression analyses found that the sample size, year of publication, country, and research type were not sources of heterogeneity; therefore, we abandoned the combined analysis. The overwhelming majority of the included literature reported that old age is a significant risk factor for POD (Table 1). Old age has been agreed upon as an independent risk factor for POD. This risk may be caused by the deterioration of the central nervous system in elderly patients, the decline in the body's resistance to stress caused by surgery, and the decline in the regulation of the immune system (Brown et al., 2016; Wang et al., 2016).

### Comorbidities

Dementia had the greatest association with the risk of delirium after TJA. Studies by Abengaña et al. (2017) suggested that psychobehavioral symptoms in dementia patients led to sleep disorders and electrolyte disturbances, which indirectly increased the susceptibility of patients to delirium. Wharton et al. (2018) found that delirium predicted aggressive behavior in patients with dementia during hospitalization and proved that there is a close relationship between the two factors. Patients who have a history of myocardial infarction, CHF, renal disease, stroke, or mental illness have a very high risk of developing POD. It is necessary for clinicians to intervene and control coexisting diseases before surgery to better prevent POD. The results of this meta-analysis showed that OSA and pulmonary disease were not significant risk factors for delirium after TJA, which is contrary to the findings of Mirrakhimov AE and Bateman BT (Grassi et al., 2001; Krenk and Kehlet, 2012; Mirrakhimov et al., 2014). Therefore, the pathogeneses of these two factors and POD still need further study. In the TKA subgroup, hypertension and OSA were risk factors for delirium after surgery, while there was no evidence that DM was a risk factor for delirium after TKA.

### Surgical factors and drug factors

In TJA, spinal anesthesia can reduce the risk of POD, while general anesthesia is a risk factor for POD. A long operation time, a long hospital stay, low albumin and hemoglobin before surgery, low hemoglobin after surgery, transfusion and blood loss are all risk factors for POD. In the TKA subgroup analysis, we found that both general anesthesia and spinal anesthesia were not risk factors for POD. In terms of drug applications, long-term use of benzodiazepines, ACEIs and beta-blockers was a risk factor for POD in patients who underwent TJA. Existing studies have shown (Kudchadkar, 2017; Mody et al., 2018) that the application of benzodiazepines is clearly associated with delirium and memory loss; therefore, limiting the use of benzodiazepines in people who are prone to delirium is recommended. Katznelson et al. (2009) found in a retrospective study that pre-operative beta-blockers increased the risk of post-operative delirium. Therefore, patients who have been taking benzodiazepines and beta-blockers for a long time need to be aware of POD and stop or change their medication if necessary. This meta-analysis found no evidence that the use of sustained-release oxycodone, ketamine, and statins increased the risk of delirium after surgery.

Several study limitations were unavoidable. First, the 25 studies included in this analysis were cohort studies, and the sample size differences between the studies were relatively large (especially in the TKA and THA subgroups), potentially leading to heterogeneity and affecting the results of the meta-analysis. Therefore, the future direction should be to design high-quality clinical randomized controlled trials against the shortcomings of this study to further reduce clinical or methodological heterogeneity, which will provide favorable support for further verifying the conclusions of this study. Second, most of the research subjects included in the literature were patients who underwent both THA and TKA, which made it difficult to extract data for hip or knee surgery alone. Third, 17 of the 25 included studies included mixed TKA and THA populations, which caused difficulties in performing subgroup analyses based on TKA and THA alone, resulting in some risk factors being excluded from subgroup analysis.

## Conclusions

This meta-analysis summarizes four categories of factors (patient factors, comorbidities, surgical factors, and drug factors) that may increase the risk for delirium after TJA. The results showed that patient factors (old age, MMSE score, education level, and alcohol abuse), comorbidities (hypertension, DM, myocardial infarction, CHF, dementia, renal disease, stroke, and history of mental illness), surgical factors (transfusion, blood loos, duration of surgery, type of anesthesia, pre-operative albumin and hemoglobin, post-operative hemoglobin, and duration of hospitalization) and drug factors (benzodiazepine, beta-blocker, and ACEI use) were risk factors for POD, which prompts us to pay attention to these factors in the clinic.

## Data availability statement

The original contributions presented in the study are included in the article/Supplementary material, further inquiries can be directed to the corresponding authors.

## Author contributions

JZ, GL, KH, and JL contributed to the study conception and design. Literature screening, data extraction, and assessment of studies quality were performed by ML, JP, and GL. The first draft of the manuscript was written by JL and JZ. JL and BH were the guarantors. All authors commented on previous versions of the manuscript, read, and approved the final manuscript.

## Funding

This work was supported by the National Natural Science Foundation of China (Nos. 81873314 and 82004386), the Natural Science Foundation of Guangdong Province (Nos. 2022A1515010385 and 2022A1515011700), the Science and Technology Planning Project of Guangzhou (No. 202102010273), the National Key Research and Development Program (2021YFC1712804), and the Project of Guangdong Provincial Department of Finance (No.[2018]8).

## Conflict of interest

The authors declare that the research was conducted in the absence of any commercial or financial relationships that could be construed as a potential conflict of interest.

## Publisher's note

All claims expressed in this article are solely those of the authors and do not necessarily represent those of their affiliated organizations, or those of the publisher, the editors and the reviewers. Any product that may be evaluated in this article, or claim that may be made by its manufacturer, is not guaranteed or endorsed by the publisher.

## Abbreviations

POD: post-operative delirium
TJA: total joint arthroplasty
RR: relative risk
SMD: standard mean difference
MMSE: mini-mental state examination
DM: diabetes mellitus
CHF: congestive heart failure
BMI: body mass index
OSA: obstructive sleep apnea
ACEI: angiotensin-converting enzyme inhibitor
THA: total hip arthroplasty
TKA: total knee arthroplasty
MOOSE: Meta-analysis of Observational Studies in Epidemiology
NOS: Newcastle-Ottawa Scale
CIs: confidence intervals.
