# Supplementary material for: Risk factors for postoperative delirium following total hip or knee arthroplasty: A meta-analysis
Source: Front Psychol. 2022 Sep 30;13:993136. doi: 10.3389/fpsyg.2022.993136 (PMC9565976; doi:10.3389/fpsyg.2022.993136)
Supplement: Supplementary file 1 [file Data_Sheet_1.docx]

**Supplementary Appendix 1: Each database retrieval strategy**

**PUBMED**

| **Search** | **Query** | **Results** |
| --- | --- | --- |
| # 1 | (((((postoperative delirium[Title/Abstract]) OR delirium[Title/Abstract]) OR acute confusional states[Title/Abstract]) OR subacute Delirium[Title/Abstract]) OR delirium of Mixed Origin[Title/Abstract]) OR mixed Origin Delirium[Title/Abstract] | 18948 |
| # 2 | (((total joint arthroplasty[Title/Abstract]) OR total joint replacemen[Title/Abstract]) OR TJA[Title/Abstract]) OR TJR[Title/Abstract] | 4453 |
| # 3 | ((((total knee arthroplasty[Title/Abstract]) OR total knee replacemen[Title/Abstract]) OR TKA[Title/Abstract]) OR TKR[Title/Abstract]) OR knee arthroplasty[Title/Abstract] | 32230 |
| # 4 | ((((total hip arthroplasty[Title/Abstract]) OR total hip replacemen[Title/Abstract]) OR THA[Title/Abstract]) OR THR[Title/Abstract]) OR hip Arthroplasty[Title/Abstract] | 57200 |
| # 5 | #2 OR #3 OR #4 | 87844 |
| # 6 | (((risk factors[Title/Abstract]) OR risk factor[Title/Abstract]) OR predictor[Title/Abstract]) OR risk[Title/Abstract] | 2663776 |
| # 7 | #1 AND #5 AND #6 | 88 |

EMBASE

| **Search** | **Query** | **Results** |
| --- | --- | --- |
| # 1 | 'postoperative delirium':ab,ti OR delirium:ab,ti OR 'acute confusional states':ab,ti OR 'subacute delirium':ab,ti OR 'delirium of mixed origin':ab,ti OR 'mixed origin delirium':ab,ti | 28666 |
| # 2 | 'total joint arthroplasty':ab,ti OR 'total joint replacemen':ab,ti OR tja:ab,ti OR tjr:ab,ti OR 'total knee arthroplasty':ab,ti OR 'total knee replacemen':ab,ti OR 'knee arthroplasty':ab,ti OR tka:ab,ti OR tkr:ab,ti OR 'total hip arthroplasty':ab,ti OR 'total hip replacemen':ab,ti OR 'hip arthroplasty':ab,ti OR tha:ab,ti OR thr:ab,ti | 103221 |
| # 3 | 'risk factor':ab,ti OR predictor:ab,ti OR 'risk factors':ab,ti OR risk:ab,ti | 3832477 |
| # 4 | #1 AND #2 AND #3 | 116 |

COCHRANE LIBRARY

| **Search** | **Query** | **Results** |
| --- | --- | --- |
| # 1 | (postoperative delirium):ti,ab,kw OR (delirium):ti,ab,kw OR (acute confusional states):ti,ab,kw OR (subacute Delirium):ti,ab,kw OR (delirium of Mixed Origin):ti,ab,kw | 4350 |
| # 2 | (total joint arthroplasty):ti,ab,kw OR (total joint replacemen):ti,ab,kw OR (TJA):ti,ab,kw OR (TJR):ti,ab,kw | 3545 |
| # 3 | (total knee arthroplasty):ti,ab,kw OR ("total knee replacement"):ti,ab,kw OR (TKA):ti,ab,kw OR (TKR):ti,ab,kw OR (knee arthroplasty):ti,ab,kw | 8969 |
| # 4 | (total hip arthroplasty):ti,ab,kw OR ("total hip replacement"):ti,ab,kw OR (THA):ti,ab,kw OR (THR):ti,ab,kw OR (hip arthroplasty):ti,ab,kw | 7365 |
| # 5 | #2 OR #3 OR #4 | 14978 |
| # 6 | (risk factors):ti,ab,kw OR ("risk-factor"):ti,ab,kw OR (risk factor):ti,ab,kw OR (predictor):ti,ab,kw OR (risk):ti,ab,kw | 291806 |
| # 7 | #1 AND #5 AND #6 | 40 |
