# Supplementary material for: Risk factors for postoperative delirium following total hip or knee arthroplasty: A meta-analysis
Source: Front Psychol. 2022 Sep 30;13:993136. doi: 10.3389/fpsyg.2022.993136 (PMC9565976; doi:10.3389/fpsyg.2022.993136)
Supplement: Supplementary file 2 [file Data_Sheet_2.docx]

**Supplementary appendix 2 The forest map of all risk factors**

**1) Patients factors**


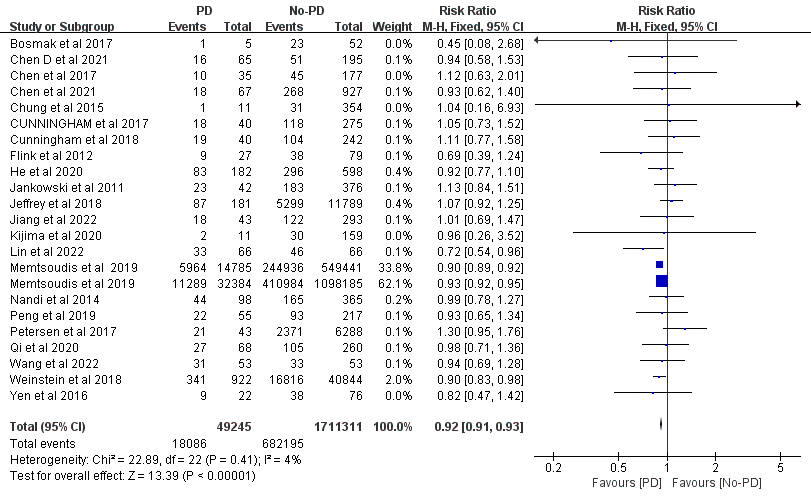


Figure S1 Male gender (TJA)


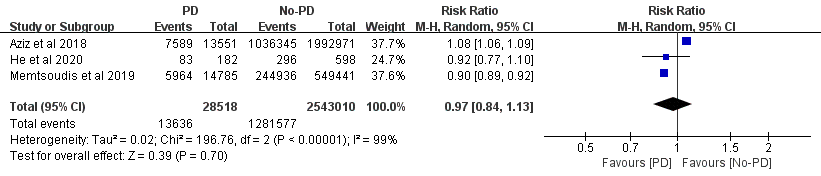


Figure S2 THA subgroup (Male gender)


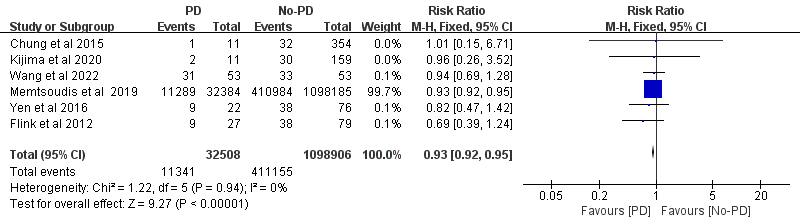


Figure S3 TKA subgroup(Male gender)


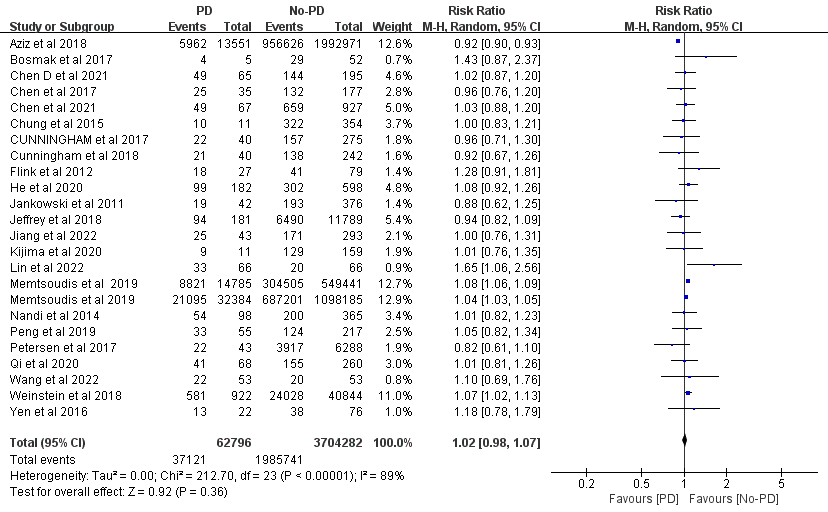


Figure S4 Female gender (TJA)


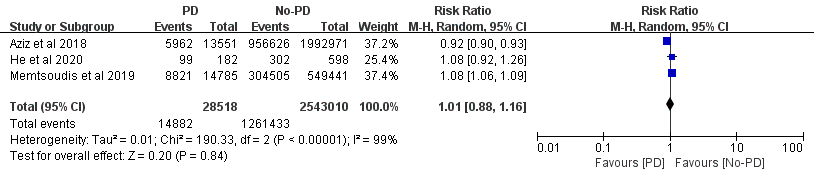


Figure S5 THA subgroup (Female gender)


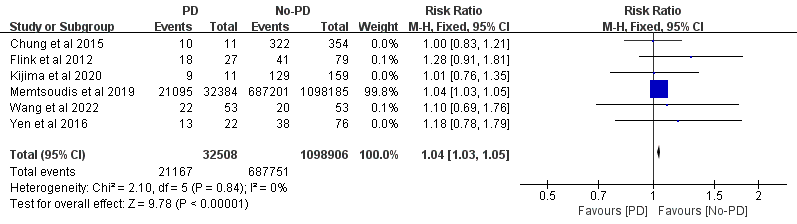


Figure S6 TKA subgroup (Female gender)


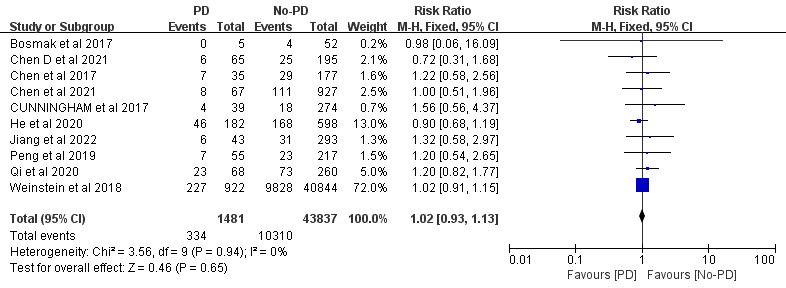


Figure S7 Smoking (TJA)


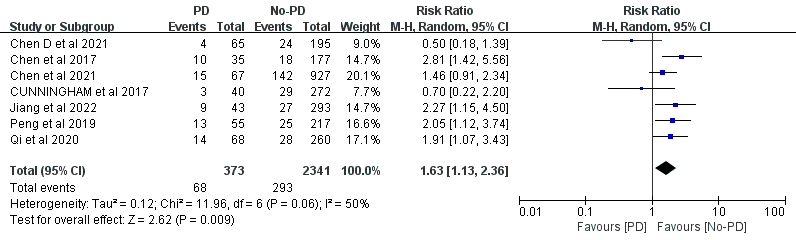


Figure S8 Alcohol abuse (TJA)


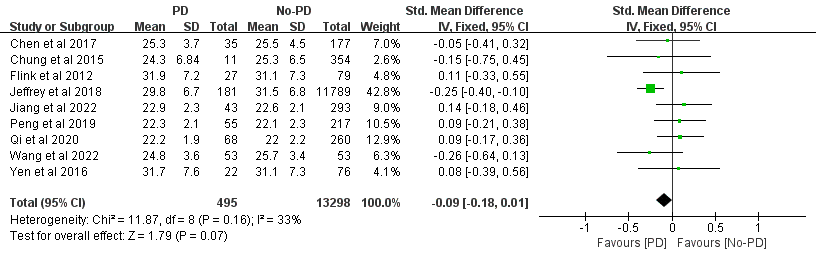


Figure S9 BMI (TJA)


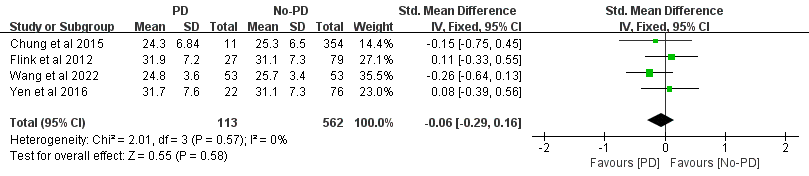


Figure S10 TKA subgroup (BMI)


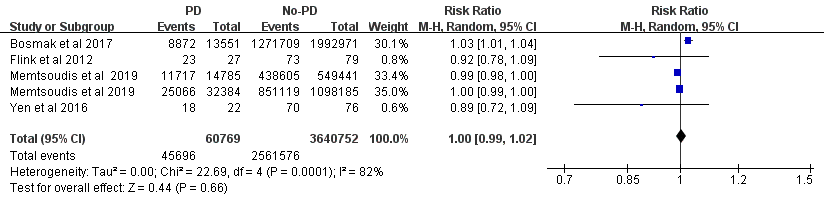


Figure S11 White race (TJA)


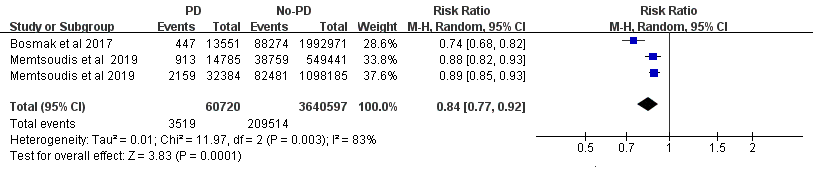


Figure S12 TKA subgroup (White race)


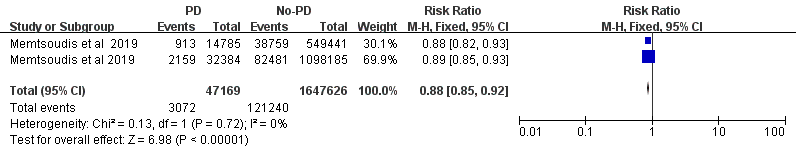


Figure S13 Black race (TJA)


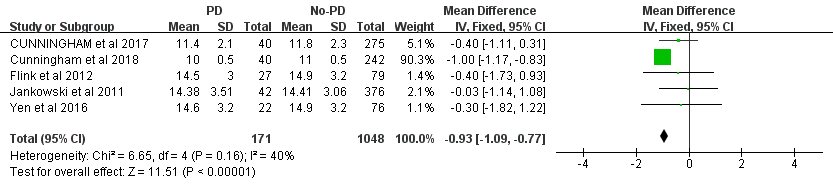


Figure S14 Length of education (TJA)


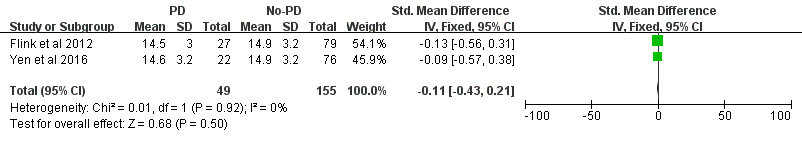


Figure S15 TKA subgroup ( Length of education)


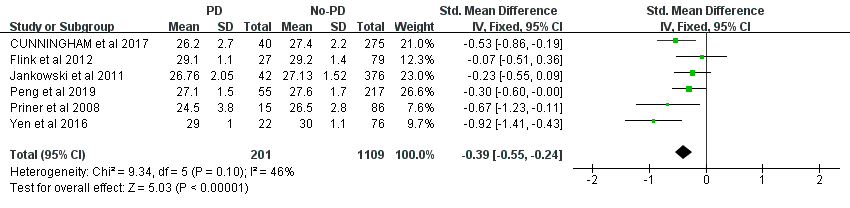


Figure S16 MMSE (TJA)


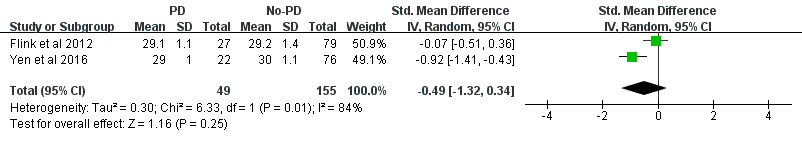


Figure S17 TKA subgroup (MMSE)

**2) Comorbidities**


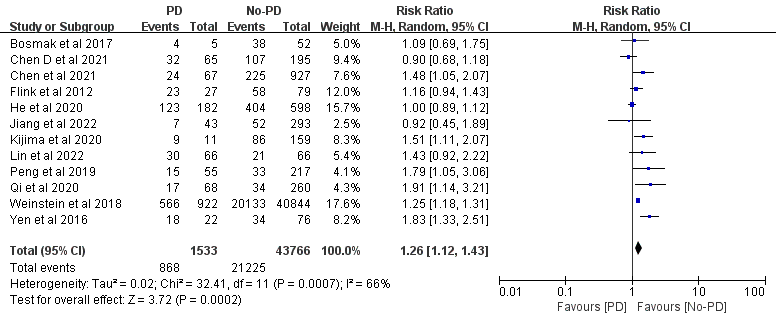


Figure S18 Hypertension (TJA)


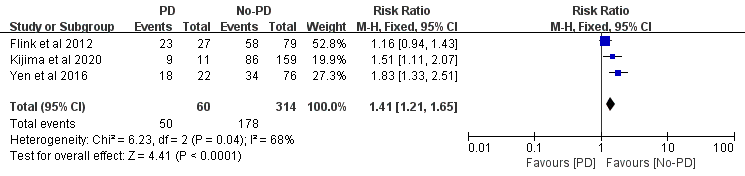


Figure S19 TKA subgroup (Hypertension)


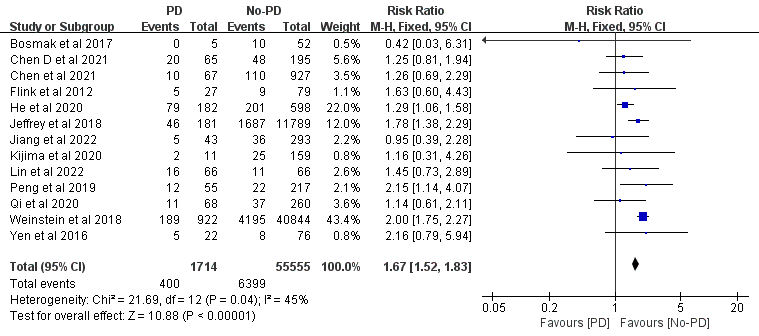


Figure S20 Diabetes mellitus (TJA)


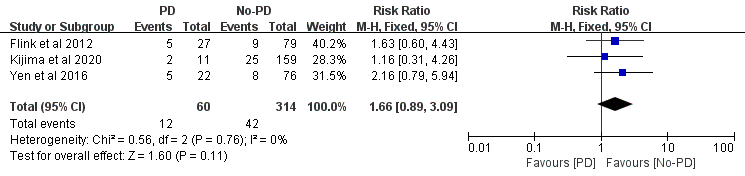


Figure S21 TKA subgroup (Diabetes mellitus)


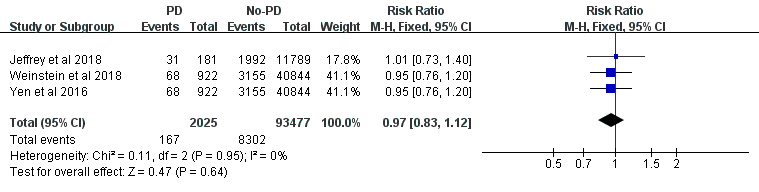


Figure S22 Obstructive sleep apnea (TJA)


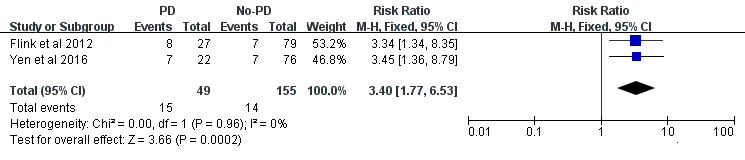


Figure S23 TKA subgroup (Obstructive sleep apnea)


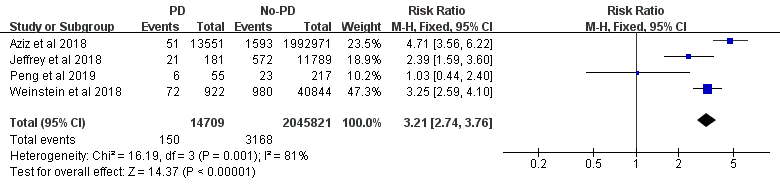


Figure S24 Myocardial infarction (TJA)


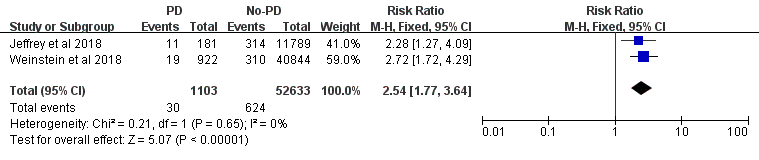


Figure S25 Congestive heart failure (TJA)


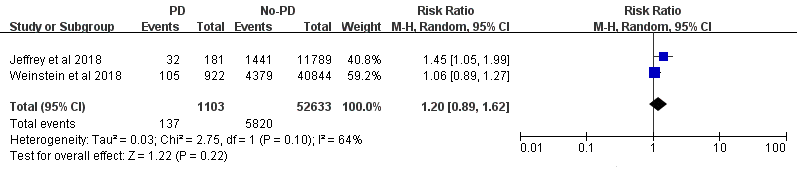


Figure S26 Pulmonary disease (TJA)


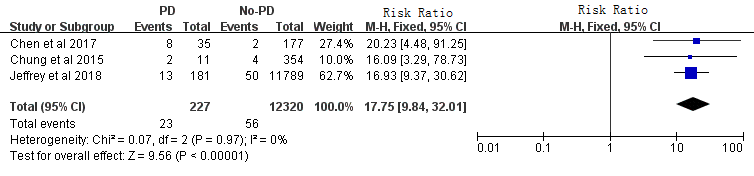


Figure S27 Dementia (TJA)


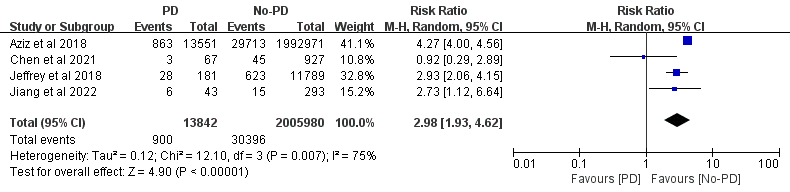


Figure S28 Renal disease (TJA)


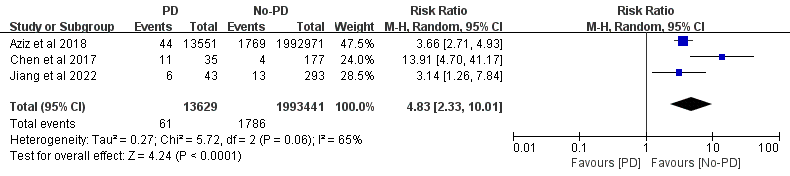


Figure S29 History of Stroke (TJA)


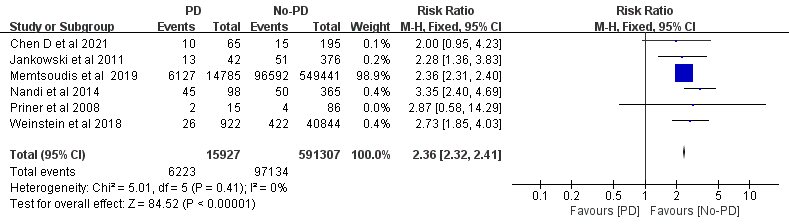


Figure S30 History of mental illness (TJA)

**3) Surgical Factors**


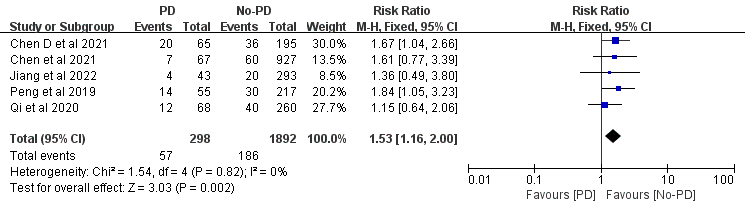


Figure S31 Transfusion (TJA)


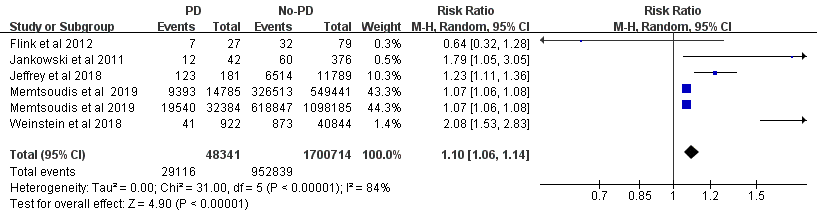


Figure S32 Type of anesthesia (general anesthesia)


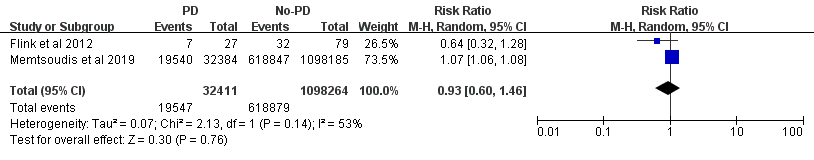


Figure S33 TKA subgroup (general anesthesia)


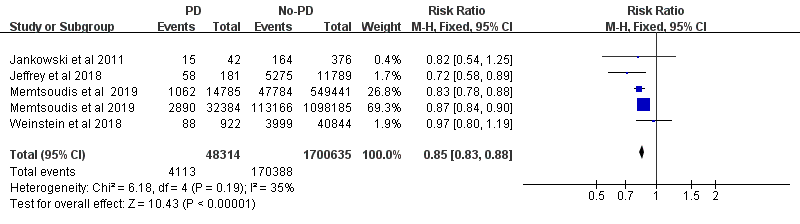


Figure S34 Type of anesthesia (spinal anesthesia)


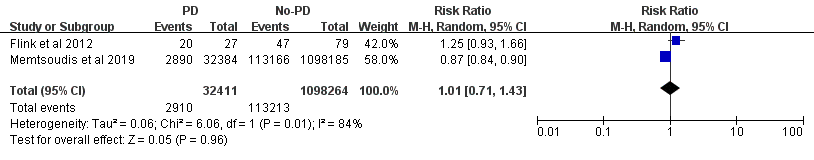


Figure S35 TKA subgroup (spinal anesthesia)


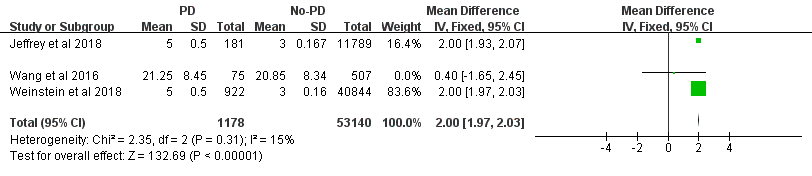


Figure S36 Duration of hospitalization


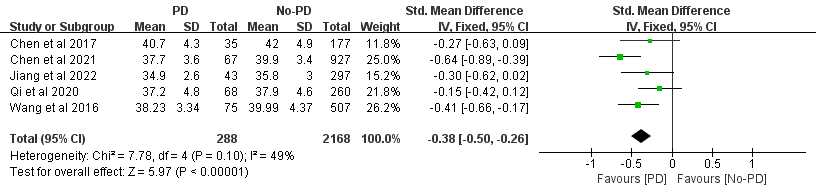


Figure S37 Preoperative Albumin (g/L)


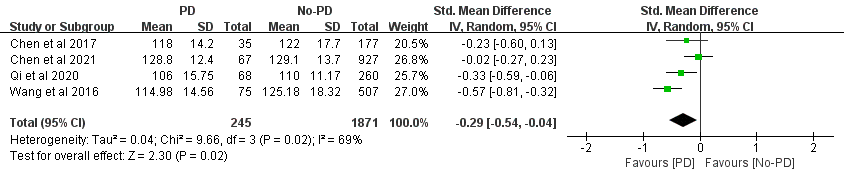


Figure S38 Preoperative Hemoglobin(g/L)


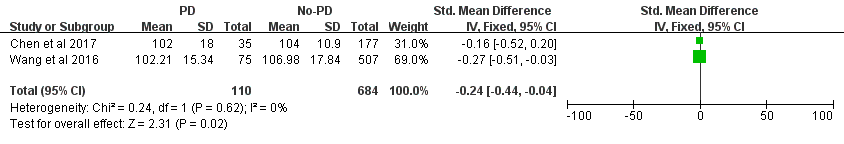


Figure S39 Postoperative Hemoglobin (g/L)


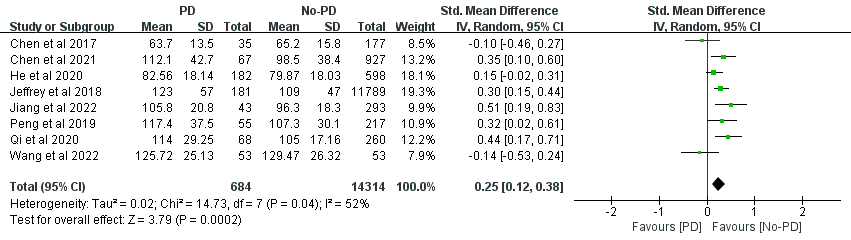


Figure S40 Duration of surgery (minutes)


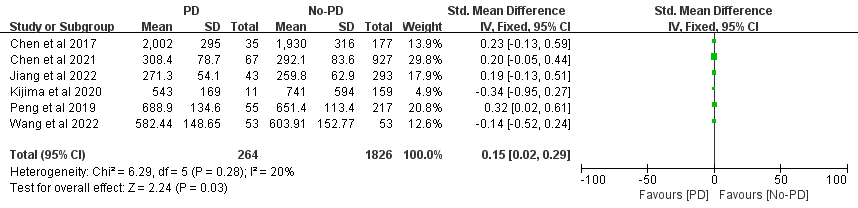


Figure S41 Total blood loss (ml)

**4) Drug Factors**


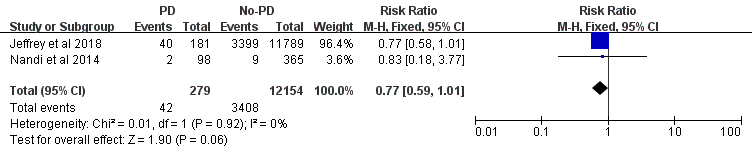


Figure S42 Sustained-release oxycodone


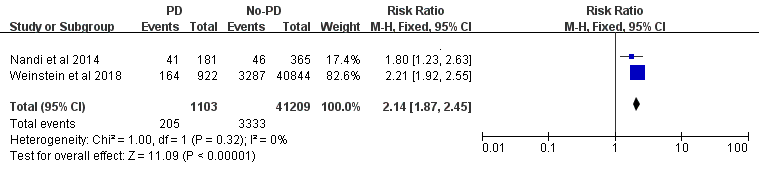


Figure S43 benzodiazepines


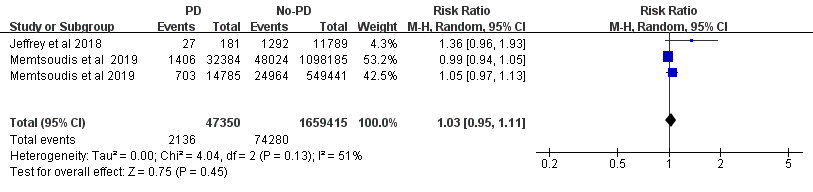


Figure S44 Ketamine


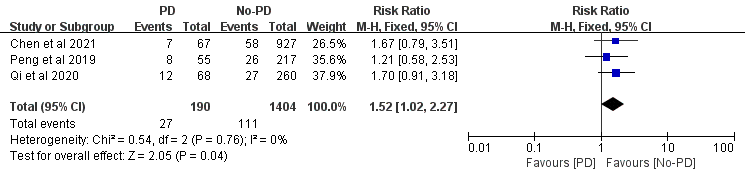


Figure S45 ACEIs


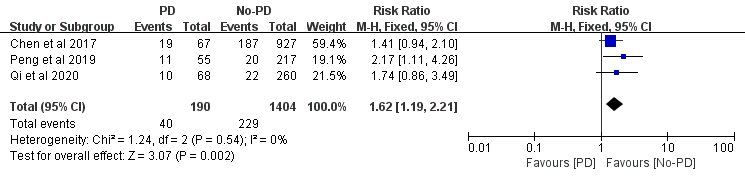


Figure S46 β-blockers


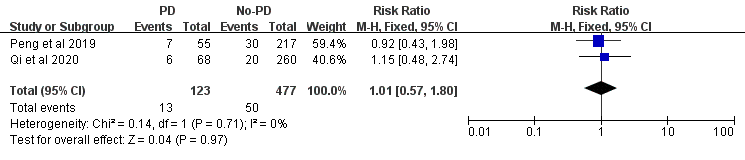


Figure S47 Statins
