# Supplementary material for: Risk factors for postoperative delirium following total hip or knee arthroplasty: A meta-analysis
Source: Front Psychol. 2022 Sep 30;13:993136. doi: 10.3389/fpsyg.2022.993136 (PMC9565976; doi:10.3389/fpsyg.2022.993136)
Supplement: Supplementary file 3 [file Data_Sheet_3.docx]

**Supplementary appendix 3 Publication Bias(Egger and Begger test)**

**1 Diabetes Mellitus**

Begg's Test

adj. Kendall's Score (P-Q) = 2

Std. Dev. of Score = 16.39

Number of Studies = 13

z = 0.12

Pr > |z| = 0.903

z = 0.06 (continuity corrected)

Pr > |z| = 0.951 (continuity corrected)

Egger's test

Std_Eff | Coef. Std. Err. t P>|t| [95% Conf. Interval]

slope | .6424208 .0870394 7.38 0.000 .4508483 .8339933

bias | -.9434223 .5150112 -1.83 0.094 -2.076954 .1901098

**2 Male gender**

Begg's Test

adj. Kendall's Score (P-Q) = -39

Std. Dev. of Score = 37.86

Number of Studies = 23

z = -1.03

Pr > |z| = 0.303

z = 1.00 (continuity corrected)

Pr > |z| = 0.316 (continuity corrected)

Egger's test

Std_Eff | Coef. Std. Err. t P>|t| [95% Conf. Interval]

slope | -.0832018 .0068363 -12.17 0.000 -.0974188 -.0689848

bias | .2321951 .2382795 0.97 0.341 -.2633343 .7277245

**3 Female**

Begg's Test

adj. Kendall's Score (P-Q) = -36

Std. Dev. of Score = 33.12

Number of Studies = 21

z = -1.09

Pr > |z| = 0.277

z = 1.06 (continuity corrected)

Pr > |z| = 0.291 (continuity corrected)

Egger's test

Std_Eff | Coef. Std. Err. t P>|t| [95% Conf. Interval]

slope | -.0475892 .0432277 -1.10 0.285 -.1380659 .0428875

bias | .4819685 .3792735 1.27 0.219 -.3118601 1.275797

**4 Smoking**

Begg's Test

adj. Kendall's Score (P-Q) = 9

Std. Dev. of Score = 11.18

Number of Studies = 10

z = 0.80

Pr > |z| = 0.421

z = 0.72 (continuity corrected)

Pr > |z| = 0.474 (continuity corrected)

Egger's test

Std_Eff | Coef. Std. Err. t P>|t| [95% Conf. Interval]

slope | .0001674 .0445511 0.00 0.997 -.1025676 .1029025

bias | .230653 .283791 0.81 0.440 -.4237702 .8850762

**5 Hypertention**

Begg's Test

adj. Kendall's Score (P-Q) = 10

Std. Dev. of Score = 14.58

Number of Studies = 12

z = 0.69

Pr > |z| = 0.493

z = 0.62 (continuity corrected)

Pr > |z| = 0.537 (continuity corrected)

Egger's test

Std_Eff | Coef. Std. Err. t P>|t| [95% Conf. Interval]

slope | .16955 .0556507 3.05 0.012 .0455525 .2935474

bias | .4958707 .7234022 0.69 0.509 -1.11597 2.107711
